# Epidemiological and clinical features, ultrasound findings and prognosis of right-sided infective endocarditis in a teaching hospital in Ouagadougou

**DOI:** 10.5830/CVJA-2013-025

**Published:** 2013-06

**Authors:** Nobila Valentin Yameogo, Kongnimisson Apoline Sondo, Aime Arsene Yameogo, Larissa Justine Kagambega, D Germain Mandi, K Jonas Kologo, Georges RC Millogo, B Jean Yves Toguyeni, Andre K Samadoulougou, N Jean-Paul Kabore, Patrice Zabsonre

**Affiliations:** Yalgado Ouedraogo Teaching Hospital, Ouagadougou, Burkina Faso; Yalgado Ouedraogo Teaching Hospital, Ouagadougou, Burkina Faso; Yalgado Ouedraogo Teaching Hospital, Ouagadougou, Burkina Faso; Yalgado Ouedraogo Teaching Hospital, Ouagadougou, Burkina Faso; Yalgado Ouedraogo Teaching Hospital, Ouagadougou, Burkina Faso; Yalgado Ouedraogo Teaching Hospital, Ouagadougou, Burkina Faso; Yalgado Ouedraogo Teaching Hospital, Ouagadougou, Burkina Faso; Yalgado Ouedraogo Teaching Hospital, Ouagadougou, Burkina Faso; Yalgado Ouedraogo Teaching Hospital, Ouagadougou, Burkina Faso; Yalgado Ouedraogo Teaching Hospital, Ouagadougou, Burkina Faso; Yalgado Ouedraogo Teaching Hospital, Ouagadougou, Burkina Faso

**Keywords:** endocarditis, right heart, venous access, Burkina Faso

## Abstract

**Introduction:**

Right-sided infective endocarditis is rare. It accounts about 5 to 10% of all infective endocarditis cases and is prevalent in patients with congenital heart disease, intravascular devices and drug addiction. Our study aimed to describe the epidemiological, clinical and echocardiographic characteristics of right-sided endocarditis and evaluate the prognosis after treatment.

**Methods:**

From January 2010 to December 2011 we recruited all patients admitted to Yalgado Ouedraogo Teaching Hospital for infective endocarditis, and selected those who had a right-sided location. The Duke criteria were used for diagnosis. We analysed entry points and underlying heart disease. The causative organisms were tracked using blood sample cultures. Ultrasound characteristics were described, and treatment and prognosis were evaluated. Patients’ follow up was conducted from recruitment to 30 June 2012.

**Results:**

In the two-year period, 14 cases of right-sided infective endocarditis were recorded, including seven cases in children. They accounted for 29.1% of all infective endocarditis cases. The mean age was 25.5 ± 12.5 years (range 9–80 years). The venous route was implicated in 12 cases (85.7%). Blood cultures were positive in 11 patients. The bacteria isolated were *Streptococcus pneumonia* in six cases, *Staphylococcus aureus* in three and *Hemophilus influenza* in two cases. HIV status was positive in three patients. Underlying heart diseases were dominated by congenital heart disease in six cases and peripartal cardiomyopathy in four others. Vegetations were located in the right heart in only 11 cases. With antibiotic treatment, a lowering of temperature was shown within an average of 10 days of follow up. Two fatalities were reported.

**Conclusion:**

This study showed that right-sided endocarditis is common in our clinical practice. This infection was prevalent in patients with congenital heart disease or peripartal cardiomyopathy in our context, and the venous route seemed to be the main entry point.

## Abstract

Infective endocarditis is a septicaemic state caused by transplantation of pathogens onto a previously healthy or injured endocardium or prosthetic valve. This definition encompasses infections developed in congenital heart disease and pacemaker probes.^1^ The valve most commonly affected is the mitral valve in 41%, followed by the aortic valve in 38%.^2^

Right-sided endocarditis is rare and represents only 5 to 10% of infective endocarditis, according to a large case series.^3^-^7^ It is found in patients with congenital heart disease or intravascular devices, and in drug addiction.^8^,^9^

The objectives of this study were to describe the epidemiological, clinical and echocardiographic characteristics of right-sided endocarditis, and assess its prognosis after treatment at the University Hospital of Ouagadougou (Burkina Faso).

## Methods

We included consecutively, from 1 January 2010 to 31 December 2011, all patients admitted to the Yalgado Ouedraogo Teaching Hospital for diagnosis of infective or probable infective endocarditis, according to the Duke criteria.^10^ Patients were recruited and admitted to the in-patient unit.

They were questioned to clarify the socio-demographic confounders (age, gender and occupation), disease history (heart disease, surgery, pacemaker, intravenous drug addiction, longterm catheter for dialysis or chemotherapy) and general signs. Physical examination collected information on the presence of signs of heart failure, modification of a pre-existing heart murmur, skin signs, Roth spots in the eye fundus examination, neurological signs, and entry points (dental, oto-rhinolaryngology, urogenital, venous access).

Para-clinical investigations focused on blood count, blood cultures, Addis count, retroviral serology, ECG, chest radiography and transthoracic echocardiography. We evaluated right ventricular systolic function by tricuspid annular plane systolic excursion (TAPSE), structural damage, and measured systolic pulmonary artery pressure using tricuspid regurgitation.

Treatment with antibiotics was administered according to the antibiogram for at least four to six weeks consecutively. We observed the evolution and possible complications during hospitalisation. On discharge from hospital, patients were examined monthly as out-patients. During these follow-up visits, we were watching for fever, dyspnoea, and signs of right heart failure or inter-current illness.

Data analysis was done with SPSS. The results are expressed as numbers and percentage.

## Results

During the study period, 48 patients with infective endocarditis were admitted to hospital, including 14 (29.1%) with right-sided endocarditis. The mean age was 25.5 ± 12.5 years (range from 9–80 years) and the gender ratio of women to men was 2.3. Children accounted for half of the right-sided endocarditis (seven cases).

A peripheral venous access had been performed in 12 patients in primary healthcare facilities and nine others had received inadequate antibiotic treatment. No case of drug addiction was recorded. Three patients were HIV positive while four were in the post-partum period.

Venous access was the entry point for bacteria in 12 patients (85.7%). The indications of venous access were malnutrition (five cases), childbirth (four cases), sickle cell crisis (two cases) and malaria (one case). In the other two cases, no entry point was found.

The clinical features included infectious syndrome in all patients, and right heart failure in nine cases. Tricuspid syndrome, consisting of fever associated with long-term lung damage (usually asymptomatic), anaemia and microscopic haematuria was found in six patients (42.8%). The diagnosis of infective endocarditis was based on the association of two major criteria in 12 patients and the association of one major criterion and three minor criteria in two other cases.

Blood sample cultures were positive in 11 patients, isolating *Streptococcus pneumonia* in six cases, *Staphylococcus aureus* in three cases and *Hemophilus influenzae* in two cases. Anaemia was common, as well as biological inflammatory syndrome (raised CRP, hyper-fibrinaemia and accelerated sedimentation rate) and leucocytosis.

**Fig. 1.**
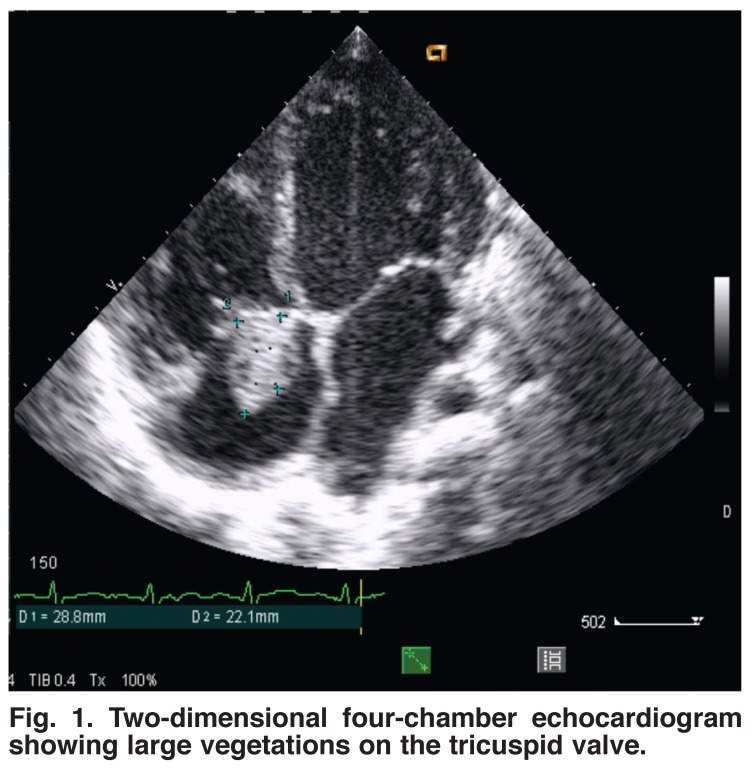
Two-dimensional four-chamber echocardiogram showing large vegetations on the tricuspid valve.

**Fig. 2.**
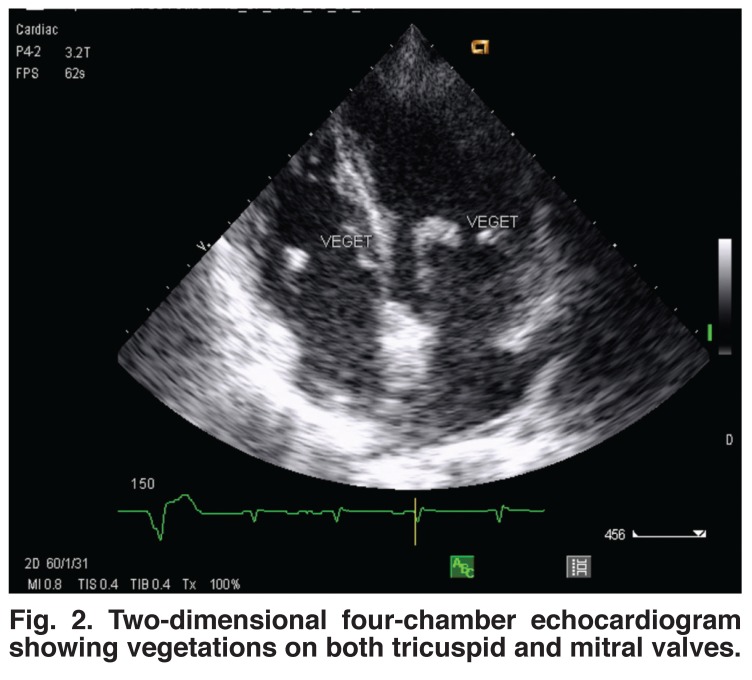
Two-dimensional four-chamber echocardiogram showing vegetations on both tricuspid and mitral valves.

**Fig. 3.**
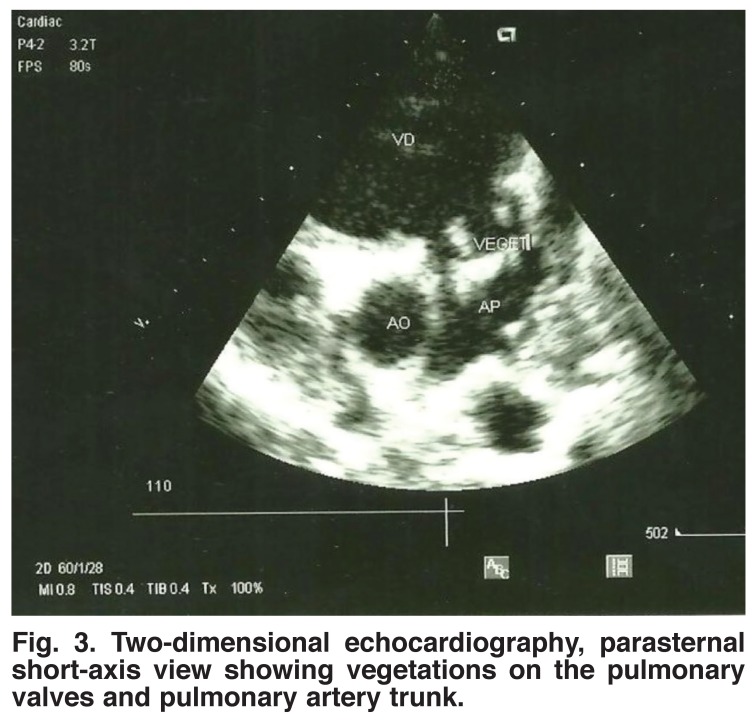
Two-dimensional echocardiography, parasternal short-axis view showing vegetations on the pulmonary valves and pulmonary artery trunk.

The electrocardiogram revealed a left atrial enlargement in seven patients, left ventricular hypertrophy in six patients, right atrial and ventricular hypertrophy in three cases and atrial fibrillation in two children. Doppler echocardiography revealed vegetations in all patients. Vegetations were localised in the right heart only in 11 cases and on the tricuspid valve only in seven cases (Fig. 1). Otherwise vegetations were found both on the tricuspid and mitral valves in two cases (Fig. 2) and on the pulmonary valves in one case (Fig. 3). The average surface area of the vegetations was 2.9 ± 0.6 cm^2^ (range 1.2–5.2). All patients had both tricuspid and pulmonary regurgitation.

Underlying heart diseases diagnosed by echocardiography Doppler are listed in Table 1.

**Table 1.** Distribution Of Underlying Heart Disease In Patients With Right-Sided Heart Endocarditis At The Yalgado Ouedraogo University Hospital From January 2010 To December 2011 (*n* = 14)

*Underlying heart disease*	*Number*	*Percentage*
Peripartal cardiomyopathy	4	28.6
Dilated cardiomyopathy	2	14.3
Ventricular septal defect	2	14.3
Pulmonary stenosis + inter-atrial communication	2	14.3
Tetralogy of Fallot	1	7.1
Restrictive ventricular septal defect + ductus arteriosus	1	7.1
No heart disease found	2	14.3
Total	14	100

Prior to the results of blood sample cultures, an early treatment with probabilistic antibiotics was made of a combination of a third-generation cephalosporin and an aminoglycoside, except in one case where the aminoglycoside was not introduced because of kidney failure. Once blood samples cultures had revealed a pathogen, the antibiotic treatment was then adjusted according to the antibiogram. Treatment was therefore adjusted in five patients. Heart failure was treated as appropriate.

The average hospital stay was 35 ± 7 days (range 24–49 days). The clinical course was marked by a lowering of temperature within an average treatment period of 10 days. Heart failure symptoms decreased as well. One fatality was reported in a child after 14 days of hospitalisation due to septic shock. After a mean follow-up period of 15.5 ± 6.8 months (range 6–30 months), a relapse occurred in two patients. Severe right heart failure was seen in three cases and one fatality was reported.

## Discussion

Right-sided location during infective endocarditis is rare. It accounts for 5–10% of cases of endocarditis, according to the literature.^2^-^6^ Right-sided endocarditis is most often reported in Africa, in very few cases. For instance, Ndiaye *et al.* in Sénégal^11^ and Compaoré *et al.* in Moroco^12^ reported six cases each. In South Africa, Naidoo *et al.* reported 15 cases.^13^ We reported 14 cases, which represented 29.1% of all infective endocarditis during the study period.

Right-sided endocarditis is most often described in drug addicts and in iatrogenic infections (catheter, post-surgery) where venous access is the point of entry for the bacteria. In fact, about 80% of tricuspid endocarditis is found in drug addicts.^14^

In our study, venous access was implicated in 85.7% of cases. Immunosuppression, which is a risk factor for right-sided endocarditis,^15^,^16^ was found in three patients in our cohort. In terms of aetiology, the microorganism most frequently isolated was *Staphylococcus aureus*.^17^

The main diagnostic tool in our study was echocardiography Doppler, by highlighting vegetations on the valves. Blood sample cultures were positive in 78.5% of cases.

Treatment of infective endocarditis is based on massive synergic long-term antibiotic therapy. The outcome is usually favourable.^17^,^19^,^20^ The prognosis of right-sided endocarditis is usually excellent with medical treatment.^17^ Complications are most often haemodynamic. They depend on the degree of valvular damage but also on underlying heart disease. Two cases of death were recorded in our study because of septic shock.

## Conclusion

This study shows that right-sided endocarditis is common in our practice. This infection is prevalent in patients with congenital heart disease or peripartum cardiomyopathy. The entry point for microorganisms is almost exclusively the intravenous route. Blood cultures can in most cases identify the bacterium. These findings require health workers to pay more attention to the maintenance of venous access in patients receiving intravenous treatment.
